# Detection of Bacterial and Viral Pathogens Using Photonic Point-of-Care Devices

**DOI:** 10.3390/diagnostics10100841

**Published:** 2020-10-19

**Authors:** Peuli Nath, Alamgir Kabir, Somaiyeh Khoubafarin Doust, Zachary Joseph Kreais, Aniruddha Ray

**Affiliations:** Department of Physics and Astronomy, University of Toledo, Toledo, OH 43606, USA; Peuli.Nath@UToledo.Edu (P.N.); MdAlamgir.Kabir@rockets.utoledo.edu (A.K.); Somaiyeh.KhoubafarinDoust@rockets.utoledo.edu (S.K.D.); Zachary.Kreais@rockets.utoledo.edu (Z.J.K.)

**Keywords:** infectious diseases, diagnostics, point-of-care devices, microfluidics, plasmonics, smartphone, lensless imaging

## Abstract

Infectious diseases caused by bacteria and viruses are highly contagious and can easily be transmitted via air, water, body fluids, etc. Throughout human civilization, there have been several pandemic outbreaks, such as the Plague, Spanish Flu, Swine-Flu, and, recently, COVID-19, amongst many others. Early diagnosis not only increases the chance of quick recovery but also helps prevent the spread of infections. Conventional diagnostic techniques can provide reliable results but have several drawbacks, including costly devices, lengthy wait time, and requirement of trained professionals to operate the devices, making them inaccessible in low-resource settings. Thus, a significant effort has been directed towards point-of-care (POC) devices that enable rapid diagnosis of bacterial and viral infections. A majority of the POC devices are based on plasmonics and/or microfluidics-based platforms integrated with mobile readers and imaging systems. These techniques have been shown to provide rapid, sensitive detection of pathogens. The advantages of POC devices include low-cost, rapid results, and portability, which enables on-site testing anywhere across the globe. Here we aim to review the recent advances in novel POC technologies in detecting bacteria and viruses that led to a breakthrough in the modern healthcare industry.

## 1. Introduction

Throughout human history, there have been several epidemics and pandemics, worldwide, due to the emergence and re-emergence of disease causing microorganisms, such as bacteria and viruses [1,2,3,4]. These pathogens cause diseases that are contagious and can be transmitted easily via aerosols, food, physical contact, body fluids of the infected person in a short period of time [5]. Infectious diseases like HIV (Human immunodeficiency virus), SARS (Severe acute respiratory syndrome), COVID-19 (coronavirus disease 2019 caused by SARS-CoV-2), influenza flu, Ebola, Herpes, Hepatitis, and tuberculosis are some of the top global health challenges at present [6,7]. For example, acquired immunodeficiency syndrome (AIDS) caused by HIV, had affected nearly 37 million people (including 1.8 million new infections) across the globe by the end of 2017 [8]. Currently, there is no cure for AIDS; early detection and prevention of transmission is the only way to defeat this disease. Some of the other pandemics include the 2009 swine flu, caused by the H1N1 Influenza virus that affected approximately 61 million people in the US alone; the 1918 Spanish flu, which resulted in the loss of almost 50 million lives worldwide [9,10]; and, more recently, COVID-19, which has infected more than 25 million people to date, with nearly ~0.8 million deaths worldwide [11,12]. The rapid spread of this infection across the continents brought life to a complete standstill. Additionally, these epidemics and pandemics have a severe impact on the economy. The COVID-19 pandemic also resulted in a severe decline of the world economy, including a 5% reduction in the GDP of the US [13]. Among bacterial infections, tuberculosis (TB) is one of the deadliest diseases caused by a bacteria *Mycobacterium tuberculosis*, which has resulted in over 1.2 million deaths in 2018 alone. It is more prevalent in countries like India, Nigeria, Indonesia, and Philippines, which account for half of the ~10 million global cases [14]. According to WHO, the estimated treatment coverage rate in 2018 was 69%, and the major challenge lies in its rapid diagnosis [14,15,16,17,18]. Foodborne pathogens, such as some virulent strains of *Escherichia coli*, are known to cause many diseases like colitis, urinary tract infections, meningitis, sepsis, and many more [19,20,21]. Every year, more than five million deaths occur worldwide due to these diseases especially in low- and middle-income countries [22]. The elderly population and children with underdeveloped immunity are particularly vulnerable to these infections. 

Thus, there has been a constant focus on early detection of these pathogens with high sensitivity and specificity, in order to prevent the spread of these infections. Some of the commonly used techniques for detection of bacteria and viruses include blood culture, high-throughput immunoassays, e.g., enzyme-linked immunosorbent assay (ELISA), polymerase chain reaction (PCR), mass spectrometry (MS), etc. [23,24,25]. Although the conventional diagnostic methods provide accurate results, they often lack sensitivity, are-time consuming, expensive, require intensive labor for sample preparation, and need trained laboratory personnel to carry out the tests. Availability of these diagnostic tests to the general population is a major issue, which has been highlighted during the COVID-19 pandemic. At present due to lack of resources, there is a strong preference to mainly test people with COVID-19 symptoms, thereby bypassing a majority of the asymptomatic population [26]. Additionally, it takes several days to get the results back from the clinic. This problem is particularly severe in developing countries, where patients usually need to travel to diagnostic centers and wait long hours in hospitals to get the test done. It has been previously reported that in developing nations over 95% deaths occur due to lack of proper diagnosis and treatment [27]. 

**Figure 1:**
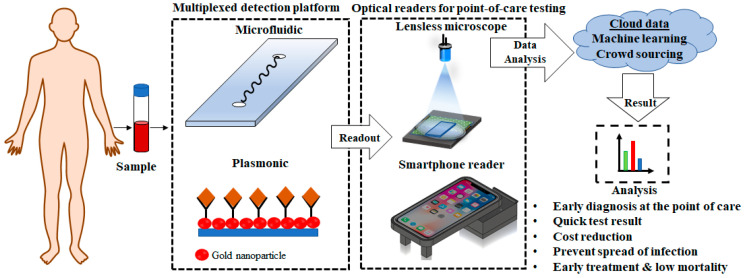
Flow diagram showing the process of sample testing using point-of care devices.

In order to address the aforementioned shortcomings, a herculean effort has been directed towards developing point-of-care (POC) devices. These devices are able to provide diagnosis at the point of care, without the need to go to a clinical laboratory [28,29,30]. An ideal POC device, such as the glucometer, can be used for testing patients at the comfort of their home, with minimal or no supervision, and should be able to provide results rapidly. These devices are designed to be low in cost, portable, and easy to use [28]. A simple POC device relies on a (i) biological recognition element (enzyme, proteins, antibody, and aptamer) that selectively interacts with the target molecules (antigen), and (ii) a transducer that monitors the interaction and provide information both qualitatively and quantitatively. Typically, the POC devices or biosensors are developed by integrating plasmonic or microfluidic devices, and an electrochemical or optical readout system into a single miniaturized platform for real-time detection of pathogens, as shown in Figure 1 [31,32,33,34]. In resource-limited settings, a POC device promises (ASSURED) Affordable, Specific, Sensitive, User-friendly, Rapid, Equipment-free analysis of immunoassays (antigen and antibody reaction) and Delivery to remote areas for ‘on-site’ analysis of samples, according to the guidelines set by WHO for developing diagnostic tools for economically underdeveloped nations, to enhance global healthcare quality [35]. 

In this review, we focus on some of the commonly used technologies utilized for developing POC devices for the detection of bacteria and viruses. These include microfluidics, plasmonics, smartphone-based imagers and lensless microscopes (Figure 1). We focus mainly on photonics-based technologies as they are capable of extremely sensitive measurement at a very high resolution and the ability to operate in multiple different modalities, e.g., colorimetric, transmission, scattering, reflection, fluorescence, interferometry, etc. Diagnosis of the diseases involves either direct detection of the pathogen or indirect detection of the antibodies produced in the body in response to a particular microorganism. We discuss some of the specific examples in detail and highlight the current state of various POC devices developed over the past decade.

## 2. Microfluidics-Based Platforms

Ever since the development of the first commercial devices µ-TAS in 1990, microfluidic technologies have evolved significantly and has been used for a large number of medical diagnostic applications [34]. Microfluidics is a field of research that deals with the manipulation of fluids at the microscale inside channels of dimension less than 1000 micron [36]. It provides the advantage of setting up experiments that require rapid diffusion, laminar flow, small sample volume, and large surface-area-to-volume ratio. The miniature size of the devices and the requirement of small sample volume makes it ideal for point of care applications. Additionally, these platforms can be used to support many different assays including immunoassays, nucleic acid amplification assays, and biochemical reactions. Therefore, microfluidics is frequently incorporated in point-of-care diagnostic devices [37,38]. 

A microfluidic (MF) platform is usually developed by using materials that are lightweight, inexpensive, portable, and disposable, such as polymers, glass, paper, and textiles, among others. Each of the materials has its own unique advantages [39,40,41,42]. For example, paper microfluidics is one of the most extensively used platforms that has been used for a variety of bio-analyte detection, due to its easy availability, low cost, biodegradability, portability, lightweight nature, and self-capillary action that eliminates the need for an external pump. The paper-based MF device is a common candidate for lateral flow immunoassays (LFIA)/test strip/rapid test/dipstick device for the detection of pathogens, antigens, and antibodies [43,44,45]. LFIAs generally consists of a sample loading pad, absorbent pad, conjugate pad, a test line, and a control line on a membrane (commonly used nitrocellulose membrane). One example of the LFIA is the recently developed tuberculosis detection, where the sample is deposited on the loading pad and flows laterally to meet the conjugate pad that contains immobilized gold nanoparticles (AuNPs) tagged with antibody (Ab) that specifically captures the CFP10-ESAT6 antigen of *M. tuberculosis* in the sample [46]. The AuNP-Ab-Antigen complex then flows along the membrane laterally due to the self-capillary action of the membrane and meets the test line, which has a second antibody that captures the AuNP-Ab-Antigen complex, resulting in a colored line. Vertical flow immunoassays (VFIA) are alternate paper-based assays that are based on the vertical flow of sample due to gravity and capillary action, and they tend to have a faster detection time [45]. In addition to papers, glass-based MF devices are also quite common due to their excellent optical properties, chemical inertness, surface stability, and solvent compatibility; thus, glass is used for fabricating devices for the detection of enzymes, antibodies, and whole cell [47]. Polymer-based MF devices fabricated by using polydimethylsiloxane (PDMS), polyethylene, polypropylene, etc., are extensively used in commercial devices due to their low cost, compared to glass and silicon, high transparency, and chemical/electrical resistance which is particularly desirable for electrochemical immunosensors [42]. Other polymers such as thermoplastics (polystyrene, cyclin olefin copolymer (COC), polyethylene terephthalate (PET), poly (methyl methacrylate) (PMMA), and polycarbonate) are also used for large scale microfluidic chip fabrication. These polymers are rigid, have good mechanical strength with no deformation issues, low water absorption, high chemical resistivity, and excellent optical properties with high UV transparency [48]. Microfluidic devices can be fabricated by using several different techniques, depending on the material. Photolithography is the common method for fabricating microfluidic devices; other methods include micromachining, plasma etching, hot embossing, injection molding, 3D printing, laser ablation, and, recently, nanofabrication [47,49]. The type of fabrication depends on the material, as well as the specific application. PDMS-based microfluidic devices are fabricated by using a soft lithography technique where liquid PDMS is poured in a micro-mold (SU-8), followed by curing at high temperature (60–80 °C) for 2 h. Meanwhile, thermoplastic polymer-based chips are fabricated in two ways: rapid prototyping and replication methods. In rapid prototyping, computer numerical controlled (CNC) machine and laser ablation techniques are employed. For large-scale production of microchannels with thermoplastic substrate, replication methods such as hot embossing, imprinting, and injection molding are commonly used. Unlike PDMS, the bonding step in thermoplastic microfluidic devices is critical. Typically, direct bonding includes thermal fusion bonding, ultrasonic bonding, and surface modification, whereas indirect bonding involves the use of chemical reagents, such as epoxy, and adhesive tape, to assist the bonding. Recent advancement in microfluidic technology includes the development of ‘hybrid devices’, i.e., integrating PDMS or paper with thermoplastic such as PDMS–PET/PDMS–PMMA [49]. 

Microfluidic platforms have been used for the detection of a variety of different pathogens that causes some of the deadliest bacterial and viral diseases such as influenza, human immunodeficiency virus (HIV), tuberculosis, Hepatitis B, Ebola, Hepatitis C, and food poisoning [50,51,52,53,54,55,56,57]. The use of microfluidic technologies is heavily featured in the newly developed POC devices for COVID-19. These include the Accula system (Mesa Biotech), which utilized the RT-PCR process; Talis One (Talis Biomedical), which is based on the loop-mediated isothermal amplification (LAMP) technology; and the Sofia 2 (Quidel), which is based on the detection of viral proteins using fluorescence [58]. Another lateral flow test for POC detection of SARS-CoV-2 was developed by combining isothermal amplification and CRISPR mediated detection method (SHERLOCK: Specific High-Sensitivity Enzymatic Reporter UnLocking) [59]. The SHERLOCK technology involves the detection of DNA or RNA by amplification of viral genome by an isothermal amplification assay and detection of the amplicon by CRISPR mediated reporter unlocking. This test called STOP (SHERLOCK Testing in One Pot) was developed to eliminate the need for sample extraction and complex reagent handling, and it can be operated at a single temperature. The best LAMP primers are designed for optimal amplification targeting N (nucleoprotein) gene. Cas12b enzyme from *Alicyclobacillus acidiphilus* (AapCas12b) was explored and operated at an optimum temperature of 55 °C for the one-pot reaction. As AapCas12b did not contain CRISPR array; 18sgRNA AacCas12b which has 97% identical sequence was combined with AapCas12b. The one-pot reaction generated faster results with higher collateral activity. The test results were generated within one hour and comparable to the standard RT-PCR technique with a limit of detection (LOD) of 100 copies of the viral genome and have been validated, using COVID-19 patient samples. 

A different microfluidic platform was developed for the detection of the influenza A (H1N1) virus by using an electrochemical approach, involving an electrochemical immunosensor coated with reduced graphene oxide (RGO) [60]. A PDMS microfluidic channel was fabricated with a thickness of 200 µm and height of 100 µm and has three electrode settings with Au-WE (gold working electrode), where the immunobinding takes place; Pt-RE (platinum reference electrode) as a stable potential reference; and Au-CE (gold counter electrode), which collects the current between WE and itself. Glass coverslips were used as a support for the three electrodes. The electrodes were coated with RGO, using dip-coating method, and, subsequently, a monoclonal Ab (mAb) specific to H1N1 virus was attached to the carboxyl group (COOH) of RGO via EDC/NHS (1-ethyl-3-(-3-dimethylaminopropyl) carbodiimide/N-hydroxysuccinimide) coupling. The binding of the H1N1 virus with the mAb attached on the electrode resulted in a voltage change, which was monitored by using cyclic voltammetry. The limit of detection (LOD) of this approach was 0.5 PFU/mL, with a linear concentration range of 1–10^4^ plaque forming unit/mL (PFU/mL), which is better than most other immunosensors developed so far. 

**Figure 2:**
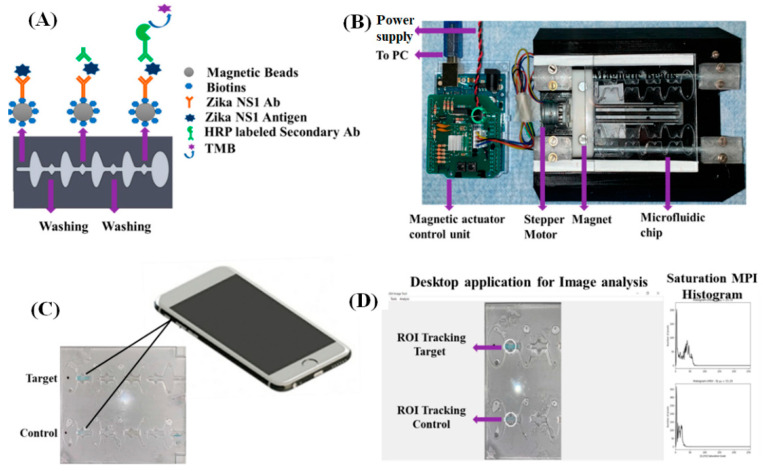
(**A**) Schematic of the enzyme-linked immunosorbent assay (M-ELISA) inside the microfluidic chip; (**B**) Magnetic actuation platform holding the microfluidic chip controlled by Arduino controller allowing bi-directional movement of the magnets; (**C**) Colorimetric changes in the chip were recorded using a smartphone; (**D**) Histogram plot of the saturation maximum pixel intensity (MPI) of the color following the M-ELISA assay on chip [62].

The detection of the Zika virus (ZIKV), which became a major global health concern in the year 2015/2016, was another challenge that was addressed using a smartphone-based fluorescent lateral flow immunoassay POC device for the detection of the non-structural protein (NS1) of ZIKV [61]. Fluorescent quantum dots (QDs) conjugated with the ZIKV NS1 antibody were used as the detection antibody in the absorption pad, mouse monoclonal ZIKV NS1 antibody in the test line as the capture antibody, and polyclonal goat anti-mouse IgG antibody in the control line of the LFIA. In presence of NS1 antigen, fluorescent QDs-ZIKV NS1 antibody captured the antigen and then flowed laterally along the nitrocellulose membrane and form QD-Ab-NS1-Ab sandwich complex on the test line. The fluorescence signal was recorded using a smartphone, under a hand-held UV lamp at 365 nm, and analyzed for quantitative detection of ZIKV NS1 antigen. The assay could detect up to 0.15 ng/mL NS1 in serum in under 20 min. Another type of automated POC microfluidic device was developed based on the colorimetric detection of ZIKV NS1 protein using ELISA assay as shown in Figure 2 [62]. A 3-layer disposable POC microfluidic chip was fabricated using polymethylmethacrylate (PMMA) and double-sided adhesive tape. The 750 µm thick top layer have inlets and outlets for sample loading. The 1.5 mm thick middle layer contained all the reagents and aqueous solution followed by the solid bottom layer which acted as the base support for the microfluidic chip. The microfluidic chip was loaded with all the reagents (Phosphate buffer, washing buffer, blocking buffer, and 3,3′,5,5′ tetramethylene blue (TMB) solution) in different chambers. The magnetic microfluidic ELISA (M-ELISA) assay involved the use of magnetic particles which were coated with biotinylated ZIKV NS1 capture antibody via neutravidin, present on the particles. The ZIKV NS1 antigen was captured using the antibody conjugated magnetic beads and transferred onto the chip. The chip was placed in a magnetic actuator platform to automatically perform washing, binding to horseradish peroxidase (HRP) tagged anti-ZIKV NS1 antibody, which completed the sandwich structure. TMB was used to generate a blue colored product and quantify the viral concentration. The magnetic actuator platform consisted of an Arduino controlling unit and a 3D printed platform that accommodated the microfluidic chip as shown in Figure 2. An iPhone X was used to capture the video, which was used for analysis based on color intensity. The limit of detection, when using this M-ELISA on-chip technique, was found to be 62.5 ng/mL in whole plasma, which is better than any other reported ELISA-based POC devices for ZIKV NS1 detection. 

Recently, loop-mediated isothermal amplification technique has been used for simple, rapid and accurate detection of positive sense single-stranded RNA virus ZIKV [63,64]. Kaarj et al. demonstrated the RT-LAMP technique on a simple disposable platform that included ‘paper microfluidics’ coupled with pH-indicator-based colorimetric assays integrated with a smartphone reader [55]. Cellulose paper, owing to its negative polarity, can be used for separating target RNA fragments from other proteins present in blood plasma of infected samples, based on their size and charge, thereby minimizing the need for pretreatment of the samples. The paper microfluidic chip was developed by using various types of material with different pore sizes, e.g., nitrocellulose (NC) paper, and grade 4 (G4) and grade 1 (G1) cellulose paper. The sample loading area (5 × 5 mm) was connected to the main channel (3 × 30 mm), where filtration occurred, and was followed by the detection zone (5 mm diameter), where the target RNA fragments were collected. After collecting the target RNA fragments the detection zone was cut out, loaded with RT-LAMP reagent mixture, sandwiched between two glass slides, and sealed with parafilm, to prevent sample evaporation. The RT-LAMP mixer had the primer and colorimetric dye, a pH indicator phenol red. The primer was designed to bind specifically to only the NS5 gene of ZIKV. The detection zone was then placed on a hot plate (68 °C) for 30 min, resulting in the amplification of the RNA, which led to a change in color from yellowish red to yellow (in the presence of the ZIKV). A smartphone-based reader was used to monitor the color change and analyze the images by using the ratio of red to green pixels. This RT-LAMP assay, using a paper microfluidic chip, can detect ZIKV with limit of detection (LOD) as low as 1 copy/µL.

HIV, which is one of the most severe global healthcare challenges over the past few decades, has attracted a lot of attention, and several commercially available rapid diagnostic tests have been developed. One of the first commercially available FDA-approved rapid diagnostics test for HIV was the Murex single-use diagnostic system (Abbott Laboratories). However, the test generated too many false results when compared to the conventional ELISA technique [65]. A similar study was conducted using five commercially available fourth and fifth generation ELISA kits for HIV detection, using different batches of confirmed number of positive and negative samples (100 in total) to evaluate the testing quality. None of the evaluated ELISA kits were able to identify all the samples correctly with 100% efficiency across all the batches, but showed high sensitivity; however, they have low specificity, particularly in the initial phases of the infection [66]. There are portable LFIA-based POC devices available in the market, such as Ora Quick Rapid in home HIV1/2 Antibody test, which can detect HIV antigen, even at low concentrations, from oral fluid, and suitable is for on-site analysis. Despite having high specificity, the test has a sensitivity of only ~92%, thereby generating few false-negative results [67]. The gold standard method for detecting HIV antigen is RT-PCR, but it can only be performed in a laboratory [68]. Recently, Phillips et al. developed a fully microfluidic rapid and autonomous analysis device (micro-RAAD) for the detection of HIV from whole-blood sample, based on loop-mediated isothermal amplification (LAMP) of HIV RNA [69]. The microfluidic device consisted of two different paper membranes: The first was the blood-separation membrane, and the second was an amplification membrane that isolated the HIV viral proteins present in blood. RNA from the isolated viral particle was amplified by using RT-LAMP reagents coated on the paper membrane which target the *gag* gene of the HIV-1 and presented the amplicons to the attached LFIA for visualization. The device was connected to a reusable temperature circuit and could be operated using a laptop or smartphone. The sensitivity of this integrated prototype was 3 × 10^5^ virus copies per reaction, or 2.3 × 10^7^ virus copies per mL of whole blood, which is comparable to clinically reported HIV-1 concentration at the peak of infection [70]. 

**Figure 3:**
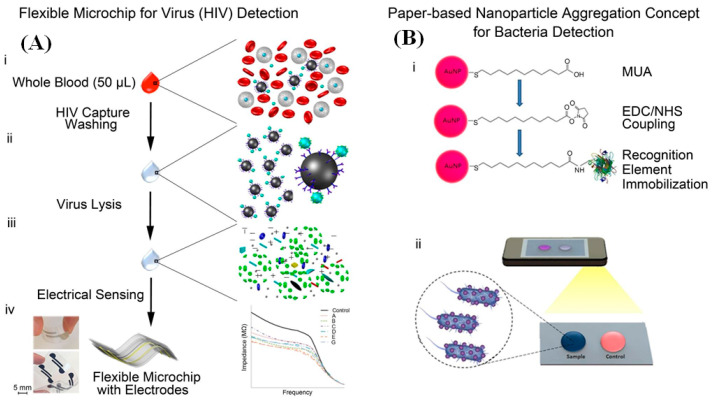
(**A**) Schematic of the flexible polyester film-based electrical sensing platform for HIV detection, including the capture of HIV through the use of anti-gp120 antibody coated magnetic beads, washing and lysis steps, and measurement of electrical impedance; (**B**) Detection of bacteria on cellulose paper, using a smartphone, based on nanoparticle aggregation assay. The following schematics depict the gold nanoparticle surface modification steps and the resultant aggregation assay which is detected by using a smartphone [71].

A different microfluidic diagnostic assay platform containing multiple detection modalities was developed by Shafiee et al., for the detection of different bio-analytes (both viruses and bacteria) from whole blood, serum, and other bodily fluids, with high specificity and sensitivity [71]. For HIV-1 detection, a microfluidic channel was fabricated by using a flexible substrate polyester film with two silver electrodes, using silver ink for detection of the virus, using viral lysate impedance spectroscopy, as shown in Figure 3A. The platform has three layers: top and bottom transparent substrate (polyester) layers and double-sided adhesive (DSA) film between the channel layer. The inlets and outlets were cut on the polyester film, with a diameter of 0.6 mm, and channels were cut on the DSA, using a laser cutter. The ink was poured through the polyester inlets, to fill the openings evenly on the DSA, using a glass coverslip. After the ink dried, the DSA was removed, leaving the electrodes on the polyester film substrate. The dimension of the electrodes was 2 mm × 1 mm. For the assay, polyclonal anti-gp120 antibody-conjugated magnetic beads were used. The HIV-1 virus was first isolated and captured by using the Ab-coated magnetic beads and detected by using the impedance magnitude measurement of the viral lysate samples. Viral lysis increased the electrical conductivity and decreases the bulk impedance magnitude of the solution. Impedance magnitude and signals were measured at 1 V with pre-evaluated frequencies between 100 Hz and 1 MHz. An electronic circuit was developed that generated an electrical response of the viral lysate in the microfluidic channel. The viral load of different subtypes was predetermined. The test samples were prepared by spiking whole blood with a cocktail of HIV-1 subtypes (A, B, C, D, E, and G). The control samples were prepared with HIV-free phosphate buffer and magnetic beads. The system could effectively detect HIV-1 virus at concentrations upwards of ~10^6^ copies/mL. 

In addition to the HIV-1, they also fabricated a paper-based nanoparticle aggregation assay system incorporated with a smartphone reader platform for detection of *E. coli* in whole blood, serum, and other bodily fluids with high specificity and sensitivity [71]. 

The device for *E. coli* detection involves the use of cellulose paper modified with nanoparticle and subsequent imaging with a smartphone as shown in Figure 3B. For *E. coli* detection, gold nanoparticle (AuNP) was modified with 11-mercaptoundecanoic acid (MUA), and succinimide groups were generated by using EDC/NHS mixture for attachment of the amine-terminated proteins on the MUA-AuNP surface. For effective binding of *E. coli* to AuNP, liposaccharide binding protein (LBP) was added to the AuNP-MUA solution. The *E. coli* spiked samples along with AuNP solution was then added to the cellulose paper, using the drop method and dried. The dried paper was then placed in a black box and illuminated with LED light. This test was based on the nanoparticle aggregation assay. The presence of *E. coli* resulted in the aggregation of the AuNPs, causing a visual color change of AuNP from red to blue. Images were captured, using a smartphone, and the individual RGB values were used for analysis. The limit of detection of this assay was reported to be 8 colony forming unit/mL (CFU/mL). 

Tuberculosis (TB) is another deadly bacterial disease that has attracted a lot of attention. Commercially available ELISA systems for the detection of tuberculosis are based on the interferon gamma release assay (IGRA). The T cells in TB infected patients produces a pro-inflammatory cytokine, interferon-gamma (IFNγ) in response to TB specific antigens, which is used for diagnosis purpose. These IFNγ producing T cells are quantified by using ELISA spot [72,73]. However, the process (IGRA) is time-consuming and requires pre-incubation of blood with TB antigens before sample preparation. Modifying this technology, Evans et al. fabricated a low-cost ELISA amperometric detection unit, using commercially available lab-on-a-chip printed circuit board (PCB) integrated with a microfluidic channel and electrodes attached to the PCB surface for the detection of cytokine IFNγ, with high sensitivity [74]. The device consists of a gold (Au) electrode sensor chip surface in the microfluidic channel that was immobilized with capture antibody anti-IFNγ Fab′(Cys)3. Samples with IFNγ was then flowed (flow rate 25 µL/min, 4 min) across the channel over the sensor chip and plasmon resonance unit (RU) spectra were recorded. The assay involves the use of 3,3′,5,5′ tetra-methylene blue (TMB) as the chromogenic substrate as it is both electrochemically and optically active molecule. TMB acts as a hydrogen donor by enzymatic reduction of hydrogen peroxide by the horseradish-peroxidase-enzyme (HRP) and hydrogen-peroxide-producing free hydroxyl ions and a blue-colored product. TMB is a colorless substrate, but the resulting product (di-imine) is bright blue in color and as the pH is lowered, a change in color from blue to yellow was observed. The absorbance, measured at 450 nm by using a nanodrop spectrophotometer, was used to quantify the change. For the electrochemical assay, fluid wells (50 µL volume) were fabricated by using polymethyl methacrylate (PMMA) over the two Au electrodes fixed to the PCB surface, and reference electrode Ag/AgCl was introduced. The change in current flow due to the electrochemical reactions at the electrode surface was measured by the in-house electronic unit. This assay was able to detect IFNγ with just 30 µL samples at concentrations ranging from 10 to 2000 pg/mL.

All the aforementioned studies demonstrated the capability of microfluidics as a powerful tool to design affordable and disposable POC devices for the detection of a broad range of bacterial and viral pathogens, with high sensitivity and specificity.

## 3. Surface-Plasmon-Resonance-Based Platforms

**Figure 4:**
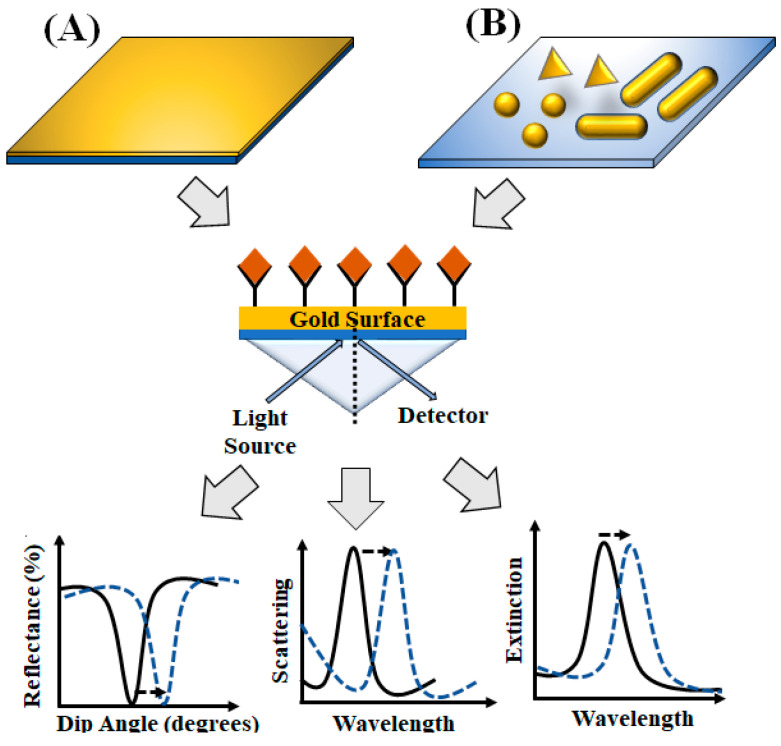
Representation of plasmon-based sensors and the different detection methods: (**A**) planar metallic thin-film-based biosensors and (**B**) localized surface plasmon resonance (LSPR)-based biosensors.

Surface plasmon resonance is another diagnostic platform that has been extensively used for the detection of viruses and bacteria [75,76]. Surface plasmons are oscillating electrons on the metallic surfaces that can be excited by shining specific wavelengths of light under certain configurations. Typically, surface plasmon resonance (SPR) is achieved by exciting electrons using evanescent waves, via total internal reflection using prisms. The propagation of evanescent waves is highly dependent on the refractive index of the material (dielectric medium) surrounding the metal surface. SPR biosensors [77,78] typically employ a thin metal film usually gold, silver or aluminum, where biorecognition elements (e.g., antibody, aptamers, etc.) are attached, and plasmons are excited on its surface by the light wave. The binding of the pathogens to the recognition element on the metallic surface leads to an increase in the refractive index of the medium, thereby changing the propagation constant of the surface plasmons. This change of refractive index is measured either by monitoring the change in the resonance angle or the shift in excitation wavelength (Figure 4). This also enables the study of binding affinity and kinetics in real time. Materials with negative real permittivity, such as gold and silver, support surface plasmon polariton (photon strongly coupled to an electric dipole) and thus show the plasmonic activity. The most commonly used material for SPR-based biosensors is gold, as it can be easily functionalized with thiol (-SH) group for surface modification and immobilize of antibodies [78]. The materials used can be of different shapes and sizes, e.g., nanoparticles (spheres, rods, and pyramids), thin films, etc. For localized surface plasmon resonance (LSPR) (Figure 4), nanoparticles smaller than the wavelength of light is typically used [79]. The use of nanoparticles effectively localizes the surface plasmons, and the evanescent wave can extend up to a few tens of nanometer into the sensing medium. In planner surface plasmon resonance (PSPR), a thin film of metal (sheet) is used instead of nanoparticles [33]. The damping of the evanescent wave is less; thus, the penetration depth becomes quite large. Therefore, a living organism can also be studied by using PSPR with a high figure of merit (FOM). However, compared to SPR, LSPR is more sensitive near the surface because of the localized field. These SPR-based techniques can be used for label-free real-time detection of analyte without external labeling (e.g., fluorescent dye, enzymes, etc.) and thus hold extreme potential in POC biosensing [80]. Another approach of utilizing SPR is by exploiting the surface-enhanced Raman scattering (SERS) signal from the target analytes. In SERS-based sensors, the analytes are adsorbed on to corrugated metallic surfaces which results in several orders of magnitude (~routinely 10^6^) enhancement of the Raman signatures from the analytes [81,82]. The enhancement of SERS signal results from the strong localization and amplification of the electromagnetic field at the hot-spots on the metallic surface [83,84,85,86,87]. These SERS-based platforms provide an alternate label-free modality for fingerprinting a range of analytes [87].

The SPR-based sensors are either stand-alone on-chip platforms, with glass or flexible polymer substrates coated with metallic films/nanoparticle, or they can be integrated with microfluidic platforms containing nanoparticles. Each has its own advantages. Combining the SPR-based sensors with microfluidics facilitates automated testing in small sample volumes [88,89,90]. Among different types of SPR-based microfluidic devices, flow through SPR sensors are quite popular, particularly in proteomics and drug discovery [90]. In this platform, an SPR sensor is integrated with continuous flow-through channels that enable the detection of multiple analytes as they flow through.

Most of the commercialized flow cell systems use a single inlet and outlet, thus only one sample can be tested at a given time. However, this can be easily addressed by using multiple microchambers for testing of several different analytes in parallel. However, the requirement of multiple valves to prevent cross flow makes it a bit complex. This platform can also be miniaturized by using portable waveguide based SPR sensors where the microfluidic channels are etched on top of the waveguide cladding. LSPR-based biosensing can also be easily performed using lateral flow test strips (discussed in Section 2), by coating nanostructures decorated with target antibodies on self-capillary flow sensor materials, like paper or membrane [33,85,86,91,92,93,94]. Digital microfluidics using electrowetting on dielectric (EWOD) is an alternate for continuous flow system where the surface property is controlled by applying a voltage and the contact angle of the droplet can be easily manipulated on the SPR sensor. This method can be easily employed for automated testing, which includes dispensing, mixing, and separating with enhanced sensitivity [90].

Another promising SPR biosensor for POC applications is based on optical fibers made of silica or polymers [89,91,94,95,96]. Light propagates inside optical fibers via total internal reflection and the evanescent field on the surface is used to excite the plasmons on the outer metallic coating. Both multi-mode and single-mode fibers can be used, but the latter has a higher sensitivity [97]. Antibodies or aptamers specific to the target antigen is conjugated to the noble metal coated on the fiber surface. The advantage of using optical fibers are manifold. Firstly, due to their flexible nature, they can be used for remote sensing applications and can be designed to operate on a small sample volume. Secondly, utilizing optical fibers reduces the complexity of the devices, by eliminating conventional optical components, thus facilitating miniaturization of the biosensors and improving its portability [97].

Plasmonic platforms have been extensively used for the detection of many pathogens [89,98,99,100,101,102,103,104,105]. The first clinically relevant nano-plasmonic POC platform for the detection and quantification of intact human immunodeficiency viruses (HIV) from unprocessed whole blood cell, with high sensitivity and specificity, was fabricated by Inci et al. [106]. The sensor was prepared by using gold nanoparticles coupled to the anti-gp120 antibody for binding the HIV. Prior to antibody conjugation, the gold nanoparticles were adsorbed onto a polystyrene surface coated with poly-L-lysine. The gold nanoparticles were coated with NeutrAvidin and the biotinylated antibody was conjugated to the particles via the biotin–avidin bond. The presence of the virus in patient blood was detected based on the shift in LSPR wavelength. This sensor can capture and quantify ~98 ± 39 copies/mL in around 1 h and could detect several subtypes of HIV in unprocessed whole blood, making it ideal for POC application.

A bio-plasmonic paper-based device (BPD) was developed for the detection of the ZIKA virus, by quantifying the amount of anti-ZIKV-nonstructural protein 1(NS1) IgG and IgM antibodies in serum [107]. The ZIKV-NS1 protein-coated gold nanorod (AuNR of length ~63 nm and diameter ~25 nm) was used to capture the antibodies. Gold nanorod was used as transducer due to its high refractive index tunability and sensitivity. ZIKV-NS1 was conjugated to the gold nanorod, using a carbodiimide crosslinker and thiol-terminated bifunctional polyethylene glycol (SH-PEG). The BPD was prepared by soaking laboratory filter paper in ZIKV-NS1 functionalized AuNRs solution. The SPR wavelength shift for the ZIKA negative serum (control, *n* = 5) was observed to be 2 nm due to nonspecific binding, whereas a shift of 7.3–8.0 nm was observed for ZIKA positive serum samples (*n* = 4). The metal–organic framework (MOF)-based preservation method rendered the device stable for a month, even at room temperature.

A different type of intensity-modulated surface plasmon resonance (IM-SPR) biosensor was developed by Chang et al. for rapid detection of avian influenza A H7N9 virus [104]. A reaction spot containing the antibody H7-mAb was used to capture the virus. The antibodies were bound to the substrate via amine coupling with the self-assembled monolayer of 11-mercaptoundecanoic acid (MUA) and 6-Mercapto-1-hexanol (MCH) (molar ratio MUA: MCH = 1:9). A reference signal from a second spot was used to quantify the test signal. A polarized light source (laser), at 635 nm, was used to illuminate the two spots and the reflected light was measured, using a data-acquisition device (DAQ). The binding of the antibody-antigen was quantified by using the change in intensity of the reflected light. This simple system was able to detect ~144 viral copies/mL in less than 10 min. 

A different approach of measuring the change in intensity due to antibody-antigen binding was demonstrated for the detection of bacteria *E. coli* [108]. In this approach the binding of the bacteria to the *E. coli* O157:H7 antibodies on the gold surface was detected by monitoring the change in photoelectric signal, associated with the SPR shift, using a linear charged coupled devices (CCD). Based on the calibration curve prepared using known quantities of bacteria, the theoretical detection limit was calculated to be 1.87 × 10^3^ CFU/mL. This sensitivity is ~4 times greater than the standard ELISA assays used to detect *E. coli* bacteria.

The change of SPR angle due to antigen–antibody binding is another approach which has been used for the detection of tuberculosis [109]. A portable SPR device was fabricated by Trzaskowski et al. by surface modification of a miniature SPR sensor Spreeta 2000 (S2k) chip with *Mycobacterium tuberculosis* (MTB) antibodies (MPT64 anti-Ag85). The SPR angle was recorded before and after adding the sample. The change in the SPR angle after the binding of MTB was used to quantify the concentration of the bacteria. The detection limit was found to be 1 × 10^4^ CFU/mL for cultured cells but in the sputum sample, it could detect secretory protein at concentration down of ~10 ng/mL. Other approaches, such as the detection of DNA fragment IS6110 using SPR, have also been used for the detection of MTB. 

Recently, an SPR-based biosensor was used to detect the SARS-CoV-2 virus. Qiu et al. developed a dual functional plasmonic biosensor combining plasmonic photothermal (PPT) effect and LSPR. The system consisted of two-dimensional gold nano-islands (AuNIs) functionalized with complementary DNA receptors for the detection of the selected sequence of the SARS-CoV-2 virus using nucleic acid hybridization technique. When the system is illuminated, a localized thermo-plasmonic heat is generated, further facilitating the nucleic acid hybridization process and enhancing the selectivity of the assay. The limit of detection of this LSPR-based detection platform was found to be ~0.22 pM [110]. Although this is a benchtop system, this technology can be translated for point-of-care application as well.

Thus, the SPR technique is a very sensitive technique that can be used to detect any bacteria or virus, with very high sensitivity, and can be integrated into any miniaturized POC device. 

## 4. Smartphone-Based Detection System

Modern smartphones with high-quality cameras and excellent computational power have the ability to perform complex analyses, making them ideal candidates for use in point-of-care (POC) devices [111]. Their ease of use and growing popularity in the modern world give smartphone credence to be used anywhere worldwide. In fact, the emerging field of smartphone-based clinical diagnostic devices has the potential to decentralize laboratory and clinical testing, as it offers practical features, such as cost-effectiveness, portability, and building connectivity between patients and healthcare providers. Modern-day smartphones are capable of detecting minute changes in optical signal resulting from any assay including immunoassays, colorimetric assays, and nucleic acid amplification. 

Advancements in different fields, such as molecular analysis, biosensors, mathematical algorithms, microfabrication, 3D-printing, and microfluidics, which occur simultaneously with the progress of cellphones and cameras, make it possible to convert a smartphone to a portable diagnostic device. Most smartphone-based diagnostic devices are designed to reduce costs and increase portability, e.g., smartphone-based microscopes and readers. The addition of an external lens, such as ball lenses, to the smartphone camera, can convert the smartphone to a brightfield microscope [112,113]. However, the curved nature of the ball-type lens can cause a distortion around the edge of the image, which can be corrected by using an objective lens and eyepiece [114]. The construction of the brightfield microscopes can be further simplified and made cost-effective by inkjet printing of lenses, using polydimethylsiloxane (PDMS) [115].

Smartphones can be also used as a fluorescent microscope which is an essential tool for modern biomedical diagnostics. A typical smartphone fluorescence microscope consists of an excitation light source (LED or laser diode), lenses and an emission filter. The wavelength of the exciting light is shorter than the emitted light and is filtered out before the detection of the fluorescent photon. Smartphones have also been used for phase-contrast imaging [116,117,118]. Similarly, spectroscopic measurements were performed by integrating dispersive elements, e.g., diffraction gratings, and pinholes or optical fibers [119]. 

**Figure 5:**
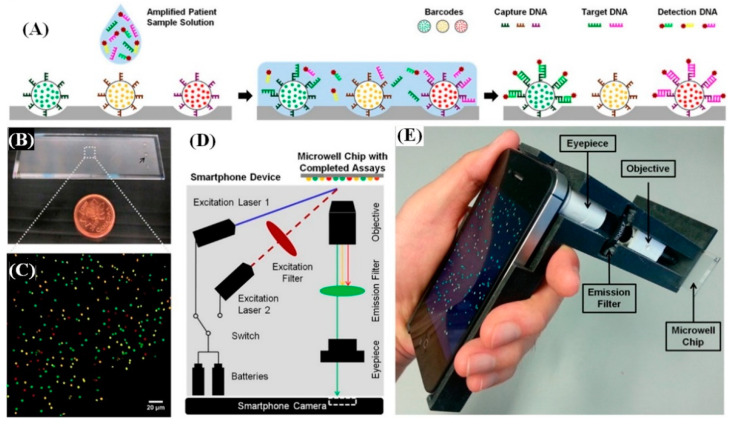
(**A**) Schematic of the fluorescence assay for detecting multiple pathogens, using a smartphone: The sample was added to a chip coated with microbeads, which are optically barcoded by quantum dots and are coated with bio-recognition element to capture a specific target molecule; (**B**) Photograph of the microwell chip containing different barcodes in each well; (**C**) Fluorescence image of the different quantum dot barcode array (Scale bar—20 µm); (**D**) Schematic of the smartphone device. Two excitation sources were used to excite the quantum dot barcoded chip independently. The optical emission is collected by a set of objective and eyepiece lenses and filtered using a long-pass filter and then imaged, using a smartphone camera; (**E**) Photograph of the smartphone device incorporated with the microwell chip. Used with permission, from Reference [131].

Over the past decade, these smartphone-based technologies have been widely used for the detection of different pathogens [57,120,121,122,123,124,125,126,127,128,129,130]. A unique fluorescence-based approach of combining quantum dot barcode technology with smartphones was used to detect HIV and Hepatitis-B virus [131]. A schematic of the assay is shown in Figure 5. The patient’s sample was first added to a chip, after amplifying the genetic target via an isothermal amplification process. The chip was composed of microbeads barcoded by different colored quantum dots. These quantum dots in turn were coated with specific recognition molecules (capture DNA). The target DNA in the sample binds to their respective capture DNA present on the microbeads. A fluorescently labeled secondary targeting agent (detection DNA) was then introduced, which specifically binds to the other end of the target DNA, thus forming a sandwich structure. The color of the fluorescence label of the detection DNA was different from the quantum dots and thus their co-localization confirmed the presence of the target viral DNA. One of the major advantages of this barcode technology is the ability to simultaneously detect multiple different viruses. A specially designed smartphone (Apple iPhone 4S) attachment containing two diode lasers (for chip illumination), a set of excitation and emission (bandpass) filters along with an objective and an eyepiece, were used as the barcode readout. The limit of detection of this assay was ~1000 viral copies/mL. 

Another smartphone-based technology capable of detecting multiple mosquito-borne viruses, i.e., Zika (ZIKV), chikungunya (CHIKV), and dengue (DENV), was developed using a loop-mediated isothermal amplification (LAMP) box [132]. The box consisted of a heating module, a housing module for the assay, a detector, and an analyzer unit to interpret the data. A heating module was used to warm the sample to about 70 °C. A dry shelf-stable assay was used containing a primer and dyes, that can be rehydrated with water and amplification buffer, prior to the assay. Furthermore, one primer was used to test three strains of ZIKV, and different primers were used for both CHIKV and DENV. Different human samples, such as urine, saliva, and blood, were spiked with the virus and then tested at various concentrations. The change in fluorescence signal due to the presence of the target virus was detected by irradiating the sample, using a 3-watt RGB LED coupled to an RGB multiband pass filter. The fluorescence images were captured by using a smartphone, and the data were analyzed, using a custom-built application (app). Furthermore, the app was also used to control the laser and heating module via a Bluetooth microcontroller.

Recently, a smartphone-based device coupled to a microfluidic chip was used for the detection of human Kaposi’s sarcoma herpesvirus 8 (KSHV) [133]. The microfluidic chip containing gold nanoparticles was coated with oligonucleotides (specific to KSHV), that aggregates in the presence of the target virus. The level of nanoparticle aggregation is proportional to the viral load and results in a change in its optical (plasmonic) properties. This change was detected by irradiating the microfluidic channel with a 520 nm LED (peak SPR wavelength) and monitoring the change in voltage across an optical sensor (photocell) placed opposite to the chip. A direct correlation was observed between the voltage drop and the optical density of the sample. The data were collected and analyzed, using a smartphone. The operating range of this device was between 500 pM and 1 μM. 

In another approach, *Mycobacterium tuberculosis* (MTBC), known to cause tuberculosis in human, was detected by using a paper-based assay and smartphone camera [134]. An array of wells was fabricated, using wax-based ink and impregnated with magnesium chloride (MgCl_2_). A solution containing gold nanoparticles functionalized with thiol-modified ssDNA oligonucleotides [135], complementary to the RNA polymerase β-subunit gene of (MTBC), was used for the detection of the MTBC. The presence of MTBC would prevent the aggregation of the gold nanoparticles due to the presence of MgCl_2_, thus preserving the red color. This change in color due to the absence of the bacteria was quantified by imaging the wells, using a mobile camera, and performing a simple RGB analysis on the images. The limit of detection of this device was reported to be 10 µg/mL MTBC sample DNA [134]. In another study, a smartphone-based fluorescence imaging platform was developed for the detection of *E. coli* in liquid samples using a sandwich immunoassay [136]. For this purpose, glass capillaries coated with anti-*E. coli* O157:H7 antibody were used to capture the *E. coli* particles in a contaminated sample. A secondary anti-*E. coli* antibody conjugated to biotin was then added in order to make a sandwich structure. The final step involved introducing streptavidin-conjugated quantum dots, which would bind to the biotin, tagged with the secondary antibodies, thereby labeling them. The capillary tubes were used to deliver the liquid into the imaging volume and served as a waveguide for the excitation light. The fluorescence emission from the quantum dots, attached to *E. coli* particles, were imaged and quantified using a cost-effective and lightweight smart-phone microscope. The detection limit of this platform was measured to be ~5 to 10 CFU/mL.

## 5. Lensless Digital Holographic Imaging

Lensless holographic imaging is another portable imaging modality that has gained prominence in the last decade due to its low cost, compactness, and wide field-of-view, which increases the throughput [93,137,138,139,140,141,142,143,144]. A lensless imaging platform is relatively simple and can be built using inexpensive light sources, e.g., LED, and a complementary metal-oxide semiconductor (CMOS) imaging sensor. A partially coherent light source is used to illuminate the sample and the resulting in-line holograms are recorded in the imaging sensor. The holograms are formed on the imaging chip due to the interference between the scattered wave from the semi-transparent sample and the transmitted wave. These holograms are digitally backpropagated to the object plane in order to reconstruct the image of the sample. Holography, being an interferometric technique, enables the extraction of both the amplitude and phase information following reconstruction. There are several approaches to reconstruct the images. One of the most commonly used technique is the angular spectrum approach, which involves multiplying the Fourier transform of the captured hologram with a transfer function $H_{z_{2}}\left(f_{x},\;f_{y}\right)$, and taking the inverse fourier transform of the product to recover the image [128]. This is expressed as follows [145]:

(1) $$E_{r}=F^{-1}\left\{F\left\{E_{i}\left(x,\;y\right)\right\}H_{z_{2}}\left(f_{x},f_{y}\right)\right\}$$

where $E_{r}$ is the reconstructed optical field of the object, $E_{i}\left(x,\;y\right)$ is the captured hologram and $H_{z_{2}}\left(f_{x},\;f_{y}\right)$ is the transfer function of the free space (*n* = 1). The transfer function is defined as [145]:

(2) $$H_{z_{2}}\left(f_{x},\;f_{y}\right)=\{\begin{array}{r} e^{ikz_{2}\sqrt{1-{\left(\frac{2\pi f_{x}}{k}\right)}^{2}-{\left(\frac{2\pi f_{y}}{k}\right)}^{2}}},\;f_{x}{}^{2}+f_{y}{}^{2}<\frac{1}{\lambda^{2}}\\ 0,\;f_{x}{}^{2}+f_{y}{}^{2}\geq \frac{1}{\lambda^{2}} \end{array}$$

Here, λ is the wavelength of the light, $k=\frac{2\pi }{\lambda }$; $f_{x}$ and $f_{y}$ are spatial frequencies; and $z_{2}$ is the sample to sensor distance. The sample to sensor distance is kept small (<1 mm), thereby leading to unit magnification. Thus, the field of view of this imaging system is quite large, compared to a conventional lens-based imaging system. The resolution of this type of imaging system is typically limited by the degree of coherence and pixel size of the CMOS sensor, but using different super-resolution techniques it was possible to achieve resolution sub-diffraction limited resolution (~250 nm) [146].

**Figure 6:**
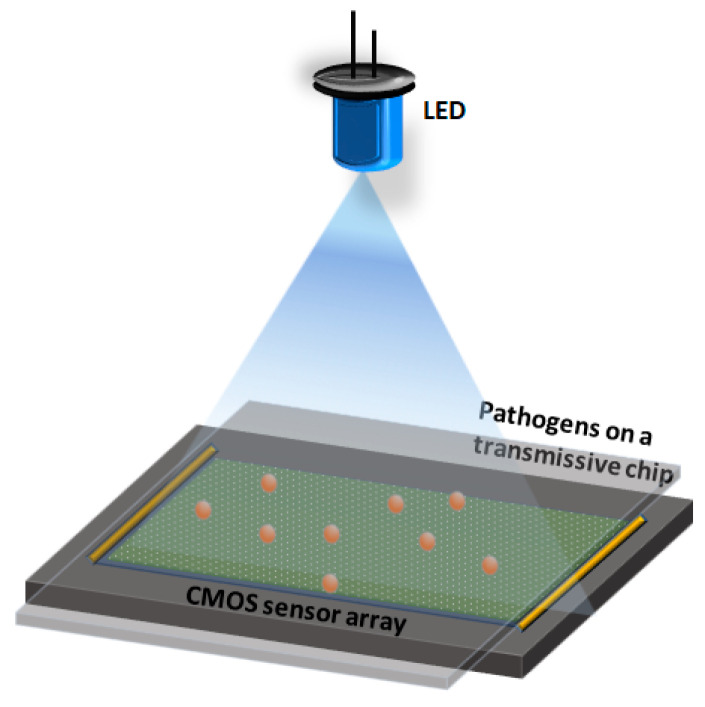
Schematic of a lensless digital holographic imaging system. A simple imaging system consists of a light source, complementary metal-oxide semiconductor (CMOS) sensor array, and a semi-transparent chip/substrate containing the sample.

The small size and low cost of these devices make them ideal for POC applications and several holographic imaging devices have been used for the detection of different types of viruses and bacteria [147,148,149,150]. In one such example, a digital lensless microscope was used to detect the Herpes Simplex Virus (HSV-1), using a microparticle clustering assay [151]. In this assay, silica microparticles (~2 µm) coated with HSV-1 antibodies were mixed with the viral particles in solution and imaged by using a holographic microscope, as shown in Figure 6. The presence of the virus caused the microparticles to aggregate, and the level of aggregation was used as the metric to infer the presence and concentration of the virus in the sample solution. Deep learning approaches were used for image reconstruction and analysis, which yielded a limit of detection as low as ~5 viral copies/µL (i.e., ~25 copies/test).

**Figure 7:**
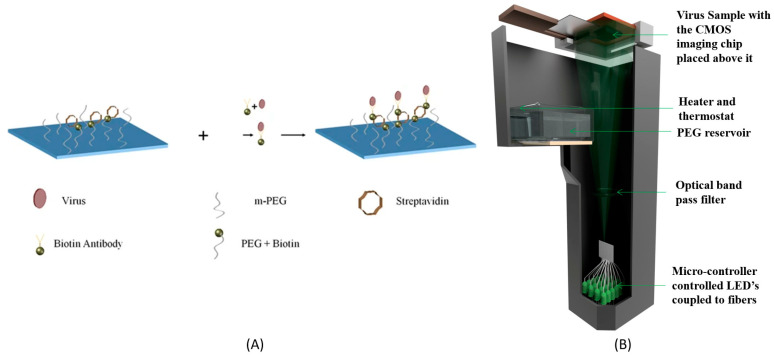
(**A**) Schematic of the HSV-1 capture assay on a specially prepared chip. (**B**) Schematic of the portable lensless microscope with pixel super-resolution capability. The device weighs less than 500 gm and is about 25 cm in height [152]. Lensless microscopy was also used to detect *Staphylococcus aureus* directly in a contact lens [154]. The contact lenses were coated with multiple layers polyelectrolytes that enables the immobilization of antibody specific to the *S. aureus* onto them. Simulated experiments were performed by incubating the antibody-coated contact lens with artificial tear fluid containing the bacteria. This was followed by the addition of a secondary antibody-coated polystyrene microparticle (5 µm), which resulted in the formation of a sandwich structure. A portable lensless microscope was used to directly image and quantify the number of microparticles present on the curved surface of the contact lens. Up to 16 bacteria/µL could be detected by using this method.

In another example, a chip for the capture of HSV-1 virus was prepared by first functionalizing the glass with silane-PEG-biotin and then adding streptavidin to it, as shown in Figure 7 [152]. Non-specific binding was eliminated by coating the glass coverslip with m-PEG-silane. The virus sample was then incubated with biotinylated antibodies specific to HSV-1. The virus-antibody-biotin was then added to the chip, in order to capture them to the surface via the biotin–avidin bond. This chip was then imaged using a lensless microscope, with pixel super-resolution capability. This was achieved by illuminating the sample sequentially with 20 different LEDs, in order to record holograms with sub-pixel shifts. These sub-pixels shifted holograms were then used to synthesize a high-resolution hologram, which was reconstructed to obtain the phase and amplitude images of the virus. Another salient feature is the use of nanolens in order to amplify the optical signature of the viral particles. Poly-ethylene glycol (PEG-400) vapor was condensed onto the chip, resulting in the formation of drop-like structures selectively around the viral particles that act as lenses. The peak phase value of the reconstructed images of the virus and nanolens was used to estimate the size of the particles and thus confirm the presence of the HSV-1 virus, which has a size of ~150–200 nm. An automated program was used to count the number of viral particles by digitally filtering out the particles with sizes outside 150–200 nm. A limit of detection of 120 viral particles per test over a field of view of ~30 mm^2^ was reported. Another approach of detecting pathogens was demonstrated by using an acoustically actuated holographic microscope, which facilitated the detection of virus (HSV-1) and bacteria (*Staphylococcus aureus*) in solution. In this approach, an acoustic transducer was coupled to a chip containing the pathogens [153]. The interdigitated transducer was used to generate surface acoustic waves which interacted with the chip to generate dispersive Lamb-type guided waves. This energy was coupled onto the liquid layer containing the pathogen and led to the formation of standing waves. The formation of the standing waves resulted in the displacement of the fluid from the antinode region, thereby exposing the pathogens and creating localized lens like liquid menisci around it. These lens-like structures (menisci) enabled the detection of *Staphylococcus aureus* bacteria and HSV-1 virus, by imaging them, using a low-cost portable holographic microscope.

## 6. Conclusions

In this review, we described some recent point-of-care technologies incorporating plasmonics, microfluidics, smartphone imagers, and lensless microscopes for simple, sensitive, rapid ‘on-site’ detection of pathogens (summarized in Table 1). Several examples covering a wide range of techniques such as immunoassays (ELISA, fluorescence, etc.) and nucleic acid amplification were discussed. Although the POC devices have been able to overcome some of the major drawbacks associated with conventional diagnostic technologies, particularly in terms of cost, throughput, and portability, there are still ways to go. A huge amount of effort needs to be dedicated in order to improve their sensitivity, specificity, ease of use, and storage, which will facilitate the use of these diagnostic techniques everywhere around the globe daily. These advanced POC devices hold the potential to revolutionize the diagnosis of the viral and bacterial pathogens, especially in resource-limited settings, thereby saving countless more lives.

## Figures and Tables

**Table 1:** A list of commonly used point-of-care (POC) technologies for the detection of some of the highly infectious bacterial and viral pathogens.

Pathogens	Detection Platform	Detection Device	Type of Assay Used	References
SARS-CoV-2	LFIA	Visual read	RT-LAMP and CRISPR	[59]
H1N1	Microfluidics	Amperometry	Electrochemical	[60]
Zika virus Zika, Dengue and Chikungunya	LFIA Microfluidics Microfluidics (Paper) Plasmonics Reaction tubes	Smartphone Smartphone Smartphone Spectral shift Smartphone	Fluorescent Immunoassay ELISA RT-LAMP Immunoassay LAMP	[61] [62] [55] [107] [132]
HIV HIV and Hep. B	LFIA Microfluidics Plasmonic Barcoded chip	Smartphone Electric sensing Spectral shift Smartphone	RT-LAMP Immunoassay Immunoassay Isothermal amplification	[69] [71] [106] [131]
H7N9	Plasmonics		Immunoassay	[104]
Kaposi sarcoma herpesvirus 8	Microfluidics	Smartphone	Nanoparticle aggregation	[133]
HSV1	Glass Chip Surface functionalized glass Chip	Lensless Holographic microscope Lensless Holographic microscope	Microparticle clustering Size-based Immunoassay	[151] [152]
*S. aureus*	Contact Lens	Holographic microscope	Immunoassay	[154]
*E. coli*	Paper microfluidic Plasmonics Glass capillaries	Smartphone CCD Smartphone	Nanoparticle aggregation Immunoassay Sandwich Immunoassay	[71] [108] [136]
*M. tuberculosis*	Microfluidic Plasmonics Paper/plasmonics	Amperometry Optical Sensor Array Smartphone	Electrochemical ELISA Immunoassay Nanoparticle aggregation	[74] [109] [134]
